# *Notes from the Field*: School-Based Surveillance of *Mycoplasma pneumoniae* Trends and Impact on School Attendance by Students and Staff Members — Missouri, Fall 2024

**DOI:** 10.15585/mmwr.mm7350a3

**Published:** 2024-12-19

**Authors:** Olivia M. Almendares, Brittney Fritschmann, Rangaraj Selvarangan, Brian R. Lee, Chris Edens, Maureen Diaz, Brett Whitaker, Shannon Tilsworth, Janelle Porter, Nibha Sagar, Jennifer E. Schuster, Jennifer L. Goldman, Hannah L. Kirking

**Affiliations:** ^1^National Center for Immunization and Respiratory Diseases, CDC; ^2^Children’s Mercy Kansas City, Kansas City, Missouri; ^3^North Kansas City School District, Kansas City, Missouri.

**Summary**

**What is already known about this topic?**

*Mycoplasma pneumoniae* is a frequent cause of respiratory illness and, in severe cases, can lead to pneumonia. Macrolide antibiotics are the recommended treatment for severe or persistent infections; macrolide resistance is uncommon in the United States.

**What is added by this report?**

School-based surveillance of *M. pneumoniae* infections at one large public Missouri school district indicated a spike in cases during fall 2024. Most (76.2%) infected students and staff members missed school or work, and approximately one half (52.4%) experienced symptoms for ≥1 week.

**What are the implications for public health practice?**

School-based surveillance can support clinical and public health decision-making, including implementation of preventive measures against respiratory illnesses in schools during the fall and winter respiratory season.

Acute respiratory illnesses (ARIs) occur frequently in school students and staff members. These infections are mostly caused by respiratory viruses; however, some are caused by bacteria, including *Mycoplasma pneumoniae*. Primarily affecting young adults and school-aged children, *M. pneumoniae* typically causes mild upper respiratory infections, but can progress to a pneumonia commonly known as “walking pneumonia” (*1*). Currently, no vaccine is available to prevent *M. pneumoniae* infection. After a period of low incidence during the COVID-19 pandemic, *M. pneumoniae* incidence increased in 2023.* Since spring 2024, CDC has reported increasing *M. pneumoniae* diagnoses in U.S. emergency departments, especially among children and adolescents (*2*). This report describes recent trends in *M. pneumoniae* activity observed in a school-based ARI surveillance program, including macrolide resistance in positive specimens. Macrolide resistance in *M. pneumoniae* infections might limit treatment options, potentially worsen patient outcomes, and facilitate the spread of resistant strains in community settings (*3*). This activity was reviewed by Children’s Mercy Hospital and CDC, deemed not research, and was conducted consistent with applicable federal law and CDC policy.^†^

## Investigation and Outcomes

Knowledge of Infectious Diseases in Schools (School KIDS) is a prospective school-based ARI and respiratory virus surveillance program in a large public Missouri school district.^§^ Participating students (prekindergarten through grade 12) and staff members could submit nasal swabs (self-collected or collected by a school nurse or parent) when they experienced one or more new ARI signs or symptoms (on-demand testing), including cough, fever, nasal congestion, shortness of breath, runny nose, wheezing, or sore throat. At the time of specimen collection, participants or parents completed a symptom survey and reported any missed days of school or work during the previous 7 days; a follow-up survey was sent 7 days after testing.^¶^ Nasal swabs were tested using multiplex polymerase chain reaction (PCR) for *M. pneumoniae*, *Chlamydia pneumoniae, Bordetella pertussis*, and eight other respiratory viruses.** *M. pneumoniae*–positive swabs were tested at CDC for the presence of mutations that confer resistance to macrolide antibiotics.

During August 18–November 6, 2024, a total of 244 samples from 226 participants in 13 of 33 schools yielded 21 *M. pneumoniae–*positive specimens, from 18 students (seven elementary, six middle school, and five high school) and three staff members. The percentage of positive test results spiked at three timepoints during this period (September 29–October 5 [15.8%], October 13–19 [22.2%], and November 3–6 [19.2%]) (Figure), similar to the increasing trends in percentages of positive test results among children aged 5–17 years nationally (*2*). The median age of students who received positive *M. pneumoniae* test results was 11 years (IQR = 8–14 years); 42.9% were female, and 85.7% identified as non-Hispanic White. In six of the 21 *M. pneumoniae*–positive specimens, other respiratory pathogens were also detected, including rhinovirus/enterovirus (five) and parainfluenza virus type 4 (one).

**FIGURE:**
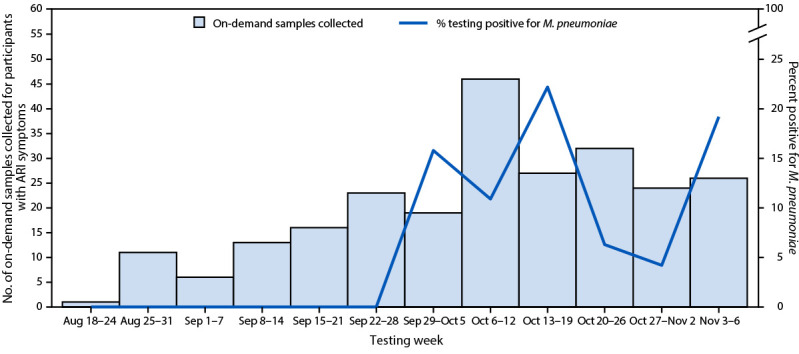
Nasal swab samples submitted for *Mycoplasma pneumoniae* testing by symptomatic* students^†^ and staff members participating in Knowledge of Infectious Diseases in Schools surveillance (N = 244) — Missouri, August 18–November 6, 2024^§^ **Abbreviation**: ARI = acute respiratory infection. * Self-report of one or more ARI signs or symptoms, including cough, fever, nasal congestion, shortness of breath, runny nose, wheezing, or sore throat. ^†^ Prekindergarten through grade 12. ^§^ Enrollment increased over time, leading to increasing numbers of samples being collected over time. On-demand testing (i.e., self-collection and submission of nasal swabs from symptomatic persons) was available to all students and staff members enrolled in Knowledge of Infectious Diseases in Schools surveillance.

All 21 participants with *M. pneumoniae–*positive test results completed both initial and follow-up surveys. In the 7 days before the *M. pneumoniae*–positive test result, frequent signs and symptoms were cough (95.2%), nasal congestion (56.5%), runny nose (52.2%), and sore throat (52.2%). After 7 days, 11 (52.4%) student and staff member participants reported the persistence of signs and symptoms. In addition, 16 participants (76.2%) missed ≥1 day of school or work, and nine (42.9%) sought medical care. Among 15 (71.4%) specimens for which macrolide susceptibility was determined, mutations known to confer resistance to macrolides in *M. pneumoniae* were detected in one specimen.

## Preliminary Conclusions and Actions

The trend in cases of *M. pneumoniae* infection detected through this school surveillance platform appears to align with national emergency department trends, particularly concerning an observed increase in cases among children and adolescents during a similar time frame (*2*). *M. pneumoniae* transmission might occur in schools, and signs and symptoms among school-aged children are similar to those caused by respiratory viruses. Preventive measures, such as handwashing, covering coughs and sneezes, and other strategies to prevent respiratory virus spread can also help reduce *M. pneumoniae* transmission in schools (*4*). Clinicians might consider testing for and treating *M. pneumoniae *infections in students or staff members with persistent or severe respiratory symptoms*.* If antibiotics are prescribed, macrolides are considered a first-line treatment for moderate to severe laboratory-confirmed infections or when clinically indicated. Second-line treatments with antibiotics, such as tetracyclines, might be considered for patients experiencing persistent or severe respiratory symptoms (*5*). Continued, voluntary school-based surveillance for *M. pneumoniae* can support clinical and public health decision-making by guiding preventive measures against ARIs in schools during fall and winter and judicious use of antimicrobials. School KIDS data raises awareness of respiratory trends, supports anticipatory guidance, and enhances clinical management, aiding decision-making across multiple levels.

## Footnotes

* The number of *M. pneumoniae* infections has varied over time, and infections might occur more frequently in summer and early fall. Cyclical peaks have been noted to occur every 3–7 years.

† 45 C.F.R. part 46.102(l)(2), 21 C.F.R. part 56; 42 U.S.C. Sect. 241(d); 5 U.S.C. Sect. 552a; 44 U.S.C. Sect. 3501 et seq.

§
https://www.frontiersin.org/journals/public-health/articles/10.3389/fpubh.2024.1408281/full

¶ Surveys were sent using Research Electronic Data Capture (REDCap) to parents or staff members based on preferred method of communication (i.e., text or email). Surveys recorded information on whether participant had acute respiratory illness signs or symptoms (i.e., cough, fever, nasal congestion, shortness of breath, runny nose, wheezing, or sore throat) and the number of days of missed school or work (if any) because of illness in the 7 days preceding completion of the survey.

** Testing was performed using QIAstat Dx respiratory panel (QIAGEN) for all pathogens noted in the report and respiratory viruses including adenovirus, human metapneumovirus, influenza A and B, parainfluenza virus types 1–4, rhinovirus/enterovirus, respiratory syncytial virus, SARS-CoV-2, and seasonal coronaviruses 229E, HKU1, NL63, and OC43.
